# Patterns and determinants of COPD-related healthcare utilization by severity of airway obstruction in Korea

**DOI:** 10.1186/1471-2466-14-27

**Published:** 2014-02-27

**Authors:** Kyungsoo Chung, Kyungjoo Kim, Jiye Jung, Kyungwon Oh, Yeonmok Oh, Sekyu Kim, Jinhee Kim, Youngsam Kim

**Affiliations:** 1 Division of Pulmonology, Department of Internal Medicine, The Institute of Chest Disease, Severance Hospital, Yonsei University College of Medicine, Seoul, Republic of Korea; 2 Yuhan Research Institute, Yongin, Gyeonggi-do, Republic of Korea; 3 Division of Health and Nutrition Survey, Korea Centers for Disease Control and Prevention, Osong, Chungcheongbuk-do, Republic of Korea; 4 Department of Pulmonary and Critical Care Medicine, and Asthma Center, Asan Medical Center, University of Ulsan College of Medicine, Seoul, Republic of Korea; 5 Department of Nursing, College of Medicine, Chosun University, Gwangju, Republic of Korea

**Keywords:** Chronic obstructive lung disease, Healthcare use, Korean National Health and Nutritional Examination Survey, National health insurance claims

## Abstract

**Background:**

We investigated patients with chronic obstructive pulmonary disease (COPD) to analyze patterns and identify determinants of healthcare use, according to the severity of airflow obstruction. We used retrospective cohort data from a combination of the 4^th^ Korea National Health and Nutritional Examination Survey (KNHANES) and Korean National Health Insurance (NHI) claims.

**Methods:**

Demographic and medical claims data were retrospectively analyzed from the 4^th^ KNHANES along with NHI claims. Eligible patients were aged ≥40 years, who underwent complete pulmonary function tests (PFTs), and had at least one inpatient or outpatient claim coded as COPD between January 1, 2007 and December 31, 2010.

**Results:**

Among 6,663 eligible participants, 897 (13.5%) had airway obstruction. Self-reported physician-diagnosed COPD comprised only 3%, and there were 870 undiagnosed COPD patients (97%). Self-reported physician-diagnosed asthma made up 3.7%. Of the 897 respondents, 244 (27.2%) used COPD-related healthcare services. The frequency of healthcare visits increased with increasing severity of airway obstruction. After a 3-year follow-up period, 646 (74.2% of those initially undiagnosed) remained undiagnosed and only 224 (25.8%) were diagnosed and treated for COPD. Only 27.5% of the 244 participants with airway obstruction who used COPD-related healthcare underwent PFTs during the study period. The percentage of prescribed medications associated with COPD increased in accordance with the severity of the COPD. Inhaled long-acting anticholinergics were prescribed for 10.9% of patients with moderate airway obstruction and for 52.4% of patients with severe obstruction. Inhaled long-acting β-agonists combined with corticosteroids were prescribed for 50% of patients with severe airway obstruction. Conversely, 44.6% of healthcare users were prescribed oral theophylline for COPD treatment, and 21.7% were also prescribed an oral corticosteroid. The determinants of COPD-associated healthcare use in respondents with obstructive lung disease were advanced age, severe airflow limitation, presence of comorbidities, and self-reported physician diagnosis of COPD.

**Conclusions:**

This study ascertained marked underdiagnosed COPD. Although the percentage of prescribed medication used to treat COPD increased with the severity of the COPD, medications primarily prescribed such as oral theophylline or oral corticosteroids are inappropriate for first-line COPD treatment.

## Background

Chronic obstructive pulmonary disease (COPD) involves progressive, irreversible airflow limitation resulting in disabling respiratory symptoms and devastating comorbidities, all of which place an increasing burden on public healthcare services [1-4]. COPD is the fourth leading cause of death, but the World Health Organization predicts that it will move up to third by 2030. Furthermore, COPD is often underdiagnosed and undertreated [5-9].

An understanding of the patterns of healthcare use is fundamental for the establishment of a healthcare plan geared towards the proper management and treatment of COPD. Data about COPD-related healthcare, such as hospital visits, the use of pulmonary function tests (PFTs), and medications prescribed according to the severity of symptoms, are lacking [10,11]. A retrospective cohort was created from the combination of data from the 4^th^ Korea National Health and Nutritional Examination Survey (KNHANES) and Korean National Health Insurance (NHI) claims. The aim of this study was to analyze patterns and identify determinants of healthcare use in COPD patients according to the severity of airflow obstruction.

## Methods

### Study population

**Figure 1:**
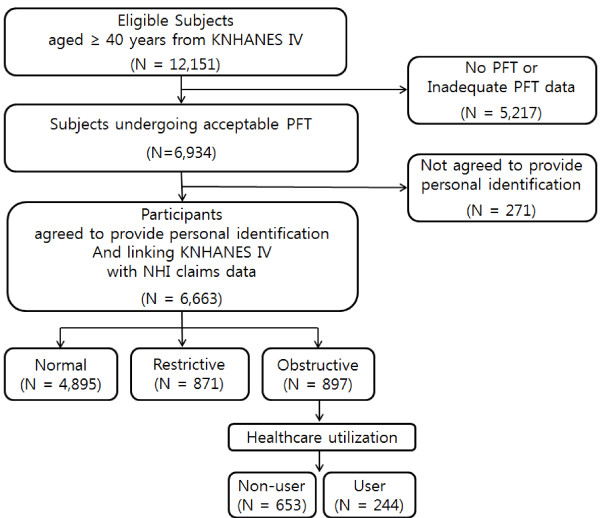
**Flow chart for selection of study participants.** PFT = pulmonary function test; Normal = FEV_1_/FVC ≥ 70% and FEV_1_ ≥ 80% predicted; Restrictive = FEV_1_/FVC ≥ 70% and FEV_1_ < 80% predicted; Obstructive = FEV_1_/FVC < 70%; Non-user = person who did not use healthcare resources at all with first-listed and secondary diagnosis of COPD; User = person who used any kind of healthcare resource with first-listed and secondary diagnosis of COPD.

Data for this study were obtained from KNHANES IV, in which a stratified, multi-stage, clustered probability design was used to select a representative sample of non-institutionalized civilians from among the Korean population. KNHANES IV data included information on demographics, smoking status, household income, education, residential area, self-reported physician diagnoses (i.e., COPD, hypertension, ischemic heart disease, osteoporosis, depression, and anemia), and PFTs. However, we inevitably included self-reported physician diagnosis of asthma in our study population because it is not possible to exclude asthma patients using self-reported physician diagnosis criteria. We describe the percentage of asthma patients in our study in Table 1. Spirometry was performed by specially trained technicians according to the 2005 American Thoracic Society/European Respiratory Society recommendations [12]. A total of 12,151 individuals from the KNHANES IV data were older than 40 years of age; however, only 57.1% of these individuals (n = 6,934) underwent complete PFTs. Informed consent was obtained from 96.1% (n = 6,663) of the individuals. Their personal information was successfully combined with records from the computerized Korean NHI claims database between January 1, 2007 and December 31, 2010. The entire population of South Korea has been provided with health coverage since 1989 by the Korean National Health Insurance System, which has managed a computerized database for all medical facilities in South Korea since 1998. The following codes from the Korean version of the International Classification of Diseases, Tenth Revision (ICD-10) were used to identify COPD-related healthcare use: simple and mucopurulent chronic bronchitis (J41), simple chronic bronchitis (J410), mucopurulent chronic bronchitis (J411), mixed simple and mucopurulent chronic bronchitis (J418), unspecified chronic bronchitis (J42), emphysema (J43), MacLeod syndrome (J430), panlobular emphysema (J431), centrilobular emphysema (J432), other emphysema (J438), emphysema, unspecified (J439), other chronic obstructive pulmonary disease (J44), chronic obstructive pulmonary disease with acute lower respiratory infection (J440), chronic obstructive pulmonary disease with acute exacerbation, unspecified (J441), other specified chronic obstructive pulmonary disease (J448), and chronic obstructive pulmonary disease, unspecified (J449). Information on COPD-related healthcare use from the NHI claims database contained the frequency of healthcare visits, the number of PFTs performed, and any prescribed medications. A total of 897 (13.4%) of 6,663 eligible individuals demonstrated an obstructive airflow pattern and were included in the final data analysis. Only 244 (27.2%) of these 897 patients used the healthcare system, and they were assessed based on disease severity (Figure 1). The study protocol was approved by the Public Institutional Review Board (PIRB 11-025-2).

**Table 1:** Baseline characteristics of study population

**Characteristics**	**Normal**	**Restrictive**	**Obstructive**	**Total**
	**No. (%)**	**No. (%)**	**No. (%)**	**No. (%)**
	**4,895 (73.5)**	**871 (13.1)**	**897 (13.4)**	**6,663 (100.0)**
Age group (yr)				
40 ~ 49	1,923 (39.3)	171 (19.6)	86 (9.6)	2,180 (32.7)
50 ~ 59	1,455 (29.7)	222 (25.5)	175 (19.5)	1,852 (27.8)
60 ~ 70	1,028 (21.0)	259 (29.7)	309 (34.5)	1,596 (24.0)
70~	489 (10.0)	219 (25.1)	327 (36.5)	1,035 (15.5)
Sex				
Male	1,834 (37.5)	409 (47.0)	628 (70.0)	2,871 (43.1)
Female	3,061 (62.5)	462 (53.0)	269 (30.0)	3,792 (56.9)
Smoking status*				
Never	3,184 (65.4)	505 (58.5)	292 (32.7)	3,981 (60.1)
Former	854 (17.5)	202 (23.4)	299 (33.5)	1,355 (20.5)
Current	830 (17.1)	156 (18.1)	301 (33.7)	1,287 (19.4)
Region of residence				
Urban	3,514 (71.8)	607 (69.7)	532 (59.3)	4,653 (69.8)
Rural	1,381 (28.2)	264 (30.3)	365 (40.7)	2,010 (30.2)
Household income (quartile)*				
1^st^ quartile	962 (20.0)	251 (29.6)	347 (40.4)	1,560 (23.9)
2^nd^ quartile	1,187 (24.7)	213 (25.1)	217 (25.3)	1,617 (24.8)
3^rd^ quartile	1,204 (25.0)	211 (24.9)	152 (17.7)	1,567 (24.0)
4^th^ quartile	1,456 (30.3)	174 (20.5)	142 (16.6)	1,772 (27.2)
Education*				
≤Elementary school	1,663 (34.1)	370 (42.8)	474 (53.4)	2,507 (37.8)
Middle school	794 (16.3)	156 (18.1)	159 (17.9)	1,109 (16.7)
High school	1,480 (30.4)	204 (23.6)	155 (17.5)	1,839 (27.8)
≥College	938 (19.2)	134 (15.5)	100 (11.3)	1,172 (17.7)
No. of co-morbidities				
0	3,125 (63.8)	429 (49.3)	477 (53.2)	4,031 (60.5)
1	1,231 (25.2)	291 (33.4)	288 (32.1)	1,810 (27.2)
2	430 (8.8)	118 (13.6)	111 (12.4)	659 (9.9)
≥3	109 (2.2)	33 (3.8)	21 (2.3)	163 (2.5)
No. of healthcare utilization				
Yes	555 (11.3)	171 (19.6)	244 (27.2)	970 (14.6)
No	4,340 (88.7)	700 (80.4)	653 (72.8)	5,693 (85.4)
Self-reported physician diagnosis of asthma				
Yes	107 (2.2%)	34 (3.9%)	33 (3.7%)	174 (2.6%)
No	4,788 (97.8%)	837 (96.1%)	864 (96.3%)	6489 (93.4%)
Self-reported physician diagnosis of COPD				
Yes	28 (0.6)	7 (0.8)	27 (3.0)	62 (0.9)
No	4,867 (99.4)	864 (99.2)	870 (97.0)	6,601 (99.1)

*The sums of some data do not equal the total because of missing data; COPD = chronic obstructive pulmonary disease; FEV_1_ = forced expiratory volume in 1 s (liter); FVC = forced vital capacity (liter).

### Definition

#### COPD and severity of airway obstruction

Subjects ≥40 years of age with FEV_1_/FVC < 70% were diagnosed with COPD. The severity of airway obstruction was classified as follows: mild (FEV_1_ ≥ 80% predicted), moderate (50 ≤ FEV_1_ < 80% predicted), and severe (FEV_1_ < 50% predicted). Additionally, normal lung function was defined as FEV_1_/FVC ≥ 70% and FVC ≥ 80% predicted. Restrictive lung disease was defined as FEV_1_/FVC > 70% and FVC < 80% predicted.

#### Users and non-users of COPD-related healthcare use

A user of COPD-related healthcare was defined as a person with a first and second diagnosis of COPD who used any kind of healthcare resource based on insurance claims data. A non-user was defined as a person with a first and second diagnosis of COPD during the follow-up period who did not use healthcare resources.

### Variables

Variables, such as the frequency of healthcare visits, performance of PFTs, and the use of prescribed respiratory medications, were used to investigate patterns of COPD-related healthcare use (presented as the annual number of visits per person). COPD-related healthcare visits were defined as any kind of visit with first and second diagnosis of COPD. Healthcare visits constituted visits to outpatient clinics, inpatient clinics, intensive care units, and emergency departments. A user of COPD-associated medication had >1 prescription for each drug category per year during the follow-up period. COPD-related medications were classified as follows: inhaled short-acting β-agonists (SABA), inhaled short-acting anticholinergics, inhaled long-acting anticholinergics (LAMA), inhaled long-acting β-agonist combined with corticosteroid (LABA/ICS), inhaled corticosteroid (ICS), oral theophylline, and oral corticosteroid. Several variables were evaluated to identify the determinants of COPD-related healthcare use in bivariate and multivariate analyses. Variables included age, sex, smoking status, residential area, household income, severity of airway obstruction, presence of comorbidities, and self-reported physician diagnosis of COPD.

### Statistical analysis

Data were analyzed using SAS software, version 9.1 (SAS Institute Inc., Cary, NC, USA). Baseline characteristics were summarized using percentages to describe categorical variables, and compared using chi-square analyses. ANOVA was used for analyzing variables associated with healthcare use among subjects classified by severity of airflow obstruction. Determinants of healthcare use between healthcare users and non-users were evaluated by logistic regression analyses, including the following covariates: age, sex, smoking status, residential area, household income, severity of airway obstruction, and physician diagnosis of COPD. Odds ratios were reported with 95% confidential intervals. A *p* value of <0.05 was regarded as statistically significant.

## Results

### Baseline characteristics of study population

Among 6,663 eligible respondents, 897 (13.5%) had airway obstruction. Of these patients, 365 (40.7%) were classified as having mild obstruction, 470 (52.4%) with moderate obstruction, and 62 (6.9%) with severe obstruction according to performance on PFTs. COPD was more prevalent in men. Self-reported physician-diagnosed COPD comprised only 3%, and self-reported physician-diagnosed asthma comprised 3.7% of the 897 subjects (Table 1).

### Patterns of healthcare use by severity of airflow obstruction

**Figure 2:**
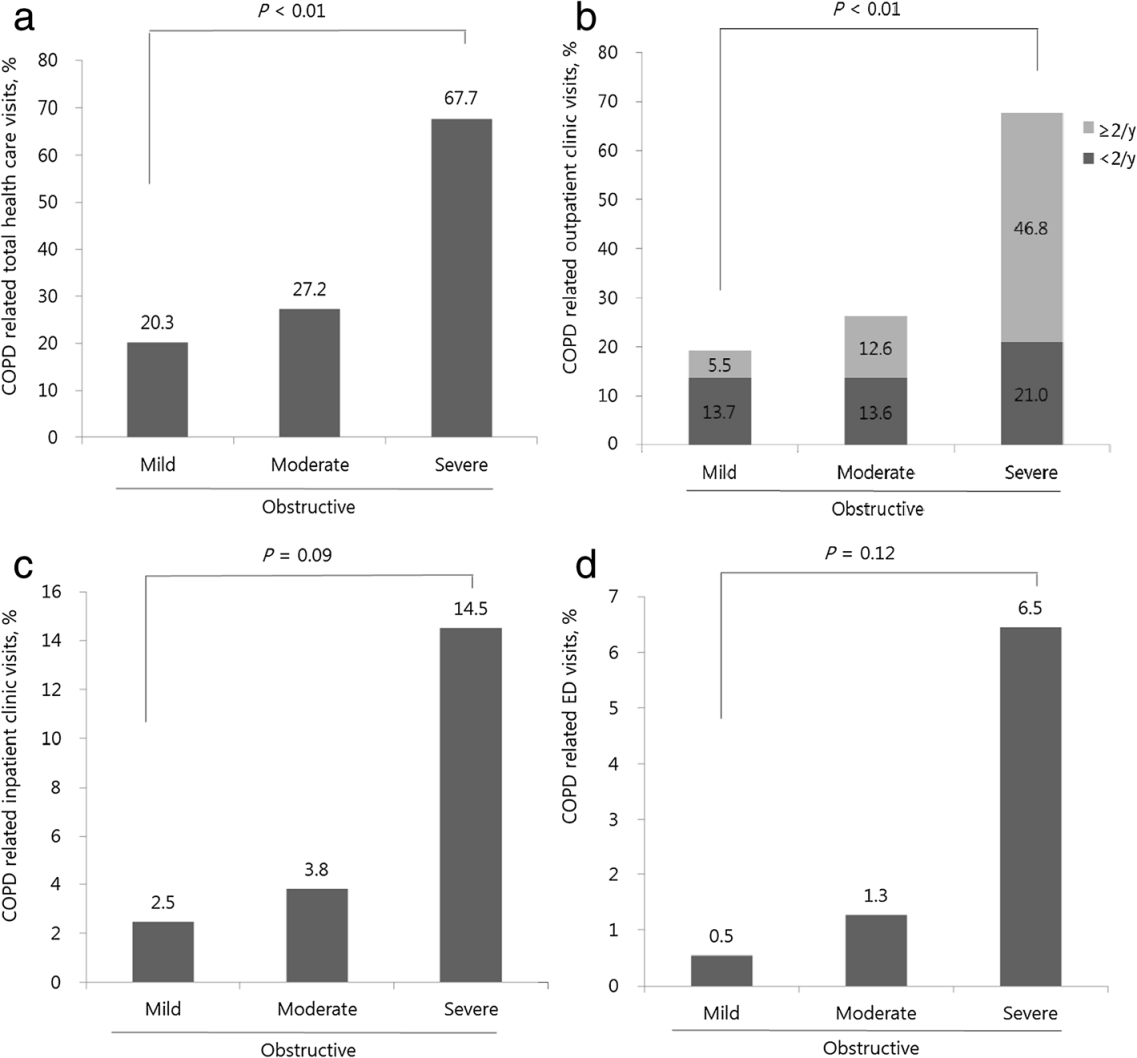
**Percentage of COPD-related healthcare visits by severity of airway obstruction.** Percentages of COPD related healthcare visits in the mild, moderate, and severe airway obstruction cohort are presented on top of each bar of the graph. Total healthcare visits were divided into outpatient clinic visits, inpatient clinic visits, and emergency department visits. **a)** COPD-related total healthcare visits, **b)** COPD-related outpatient clinic visits, **c)** COPD-related inpatient clinic visits, **d)** COPD-related emergency department visits; mild = FEV_1_/FVC < 70% and FEV_1_ ≥ 80% predicted; moderate = FEV_1_/FVC < 70% and 50 ≤ FEV_1_ < 80% predicted; severe = FEV_1_/FVC < 70% and FEV_1_ < 50% predicted; normal = FEV_1_/FVC ≥ 70% and FEV_1_ ≥ 80% predicted; restrictive = FEV_1_/FVC ≥ 70% and FEV_1_ < 80% predicted.

**Figure 3:**
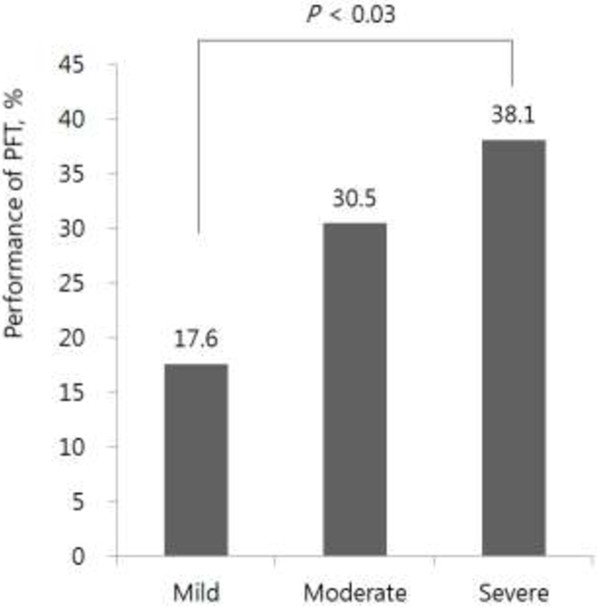
**Performance in PFTs stratified by severity of airway obstruction.** Percentages of each PFT performance in the mild, moderate, and severe airway obstruction cohort are presented on top of each bar in the graph.

Of the 897 respondents, 244 (27.2%) used COPD-related healthcare services. The frequency of healthcare visits increased with increasing severity of airflow obstruction (*p* < 0.01). Subjects with severe COPD visited healthcare facilities most frequently (Figure 2). Among those patients with airflow limitation (N = 897), there were initially 870 (97%) undiagnosed COPD patients and 27 (3%) self-reported physician-diagnosed COPD patients. After the 3-year follow-up period, 74.2% (N = 646) remained as undiagnosed COPD patients and only 25.8% (N = 224) were diagnosed and treated as a COPD, among initially undiagnosed COPD patients (N = 870). Seven patients were untreated among initially self-reported diagnosed COPD patients (N = 27). Only 27.5% of the 244 participants with airway obstruction who used COPD-related healthcare underwent PFTs during the study period (data not shown). The percentage of patients undergoing spirometry increased according to the severity of the airflow obstruction (*p* = 0.03) (Figure 3; Table 2).

**Table 2:** Patterns of healthcare visits, PFTs, and prescribed medications according to severity of airflow obstruction

	**Mild**	**Moderate**	**Severe**	
	**No. (%)**	**No. (%)**	**No. (%)**	
	**74 (30.3)**	**128 (52.5)**	**42 (17.2)**	***P *** **value**
Healthcare visit (frequency/person/year)*				
Total	2.014 ± 0.323	3.675 ± 0.473	5.764 ± 0.783	<0.001
Outpatient clinic	1.895 ± 0.325	3.594 ± 0.472	5.516 ± 0.732	<0.001
Inpatient clinic	0.118 ± 0.051	0.082 ± 0.021	0.248 ± 0.114	0.091
ICU	0.025 ± 0.020	0.005 ± 0.004	0.039 ± 0.024	0.219
ED	0.009 ± 0.007	0.023 ± 0.010	0.071 ± 0.049	0.124
PFT (frequency/person/year)*	0.109 ± 0.032	0.168 ± 0.025	0.263 ± 0.059	0.031
Medications (frequency/person/year)*				
SABA	0.050 ± 0.026	0.287 ± 0.135	0.957 ± 0.290	0.003
SAMA	0.036 ± 0.023	0.036 ± 0.015	0.200 ± 0.104	0.012
LAMA	0.178 ± 0.163	0.213 ± 0.080	1.940 ± 0.492	<0.001
LABA+ICS	0.054 ± 0.046	0.408 ± 0.137	1.280 ± 0.395	<0.001
ICS	0.050 ± 0.045	0.032 ± 0.014	0.194 ± 0.107	0.051
Oral theophylline	0.484 ± 0.121	1.156 ± 0.254	3.162 ± 0.571	<0.001
Oral corticosteroid	0.143 ± 0.081	0.390 ± 0.116	1.422 ± 0.451	<0.001

*= mean ± standard error; ICU = intensive care unit; ED = emergency department; PFT = pulmonary function test; SABA = inhaled short-acting β agonist; SAMA = inhaled short-acting anticholinergic; LAMA = inhaled long-acting anticholinergic; LABA+ICS = inhaled long-acting β-agonist combined with corticosteroid; ICS = inhaled corticosteroid.

### Patterns of prescribed medications according to the severity of airflow obstruction

**Figure 4:**
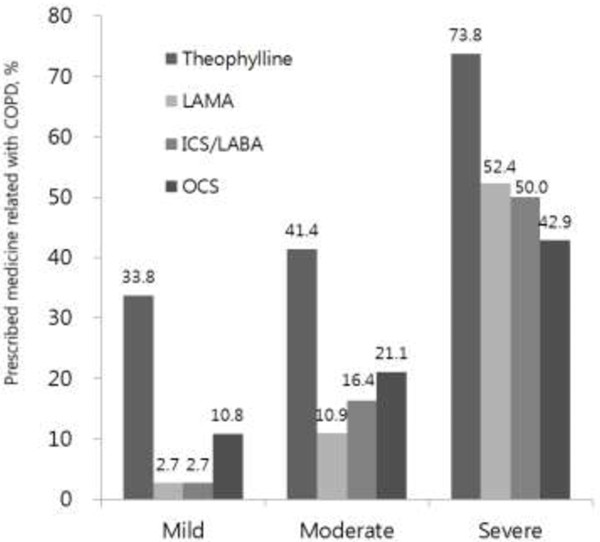
**Percentages of medications prescribed, classified by severity of airway obstruction.** Percentages of prescribed medications in the mild, moderate, and severe airway obstruction cohort are presented on top of each bar in the graph. LAMA = inhaled long-acting anticholinergic; ICS = inhaled corticosteroid; LABA = inhaled long-acting β-agonist; OCS = oral corticosteroid.

The percentage of prescribed medications associated with COPD increased in accordance with the severity of the COPD. Oral theophylline was prescribed as COPD treatment for 44.6% of healthcare users, and 21.7% of users were also prescribed an oral corticosteroid (data not shown). The percentages of prescribed oral theophylline were 33.8% for mild obstruction, 41.4% for moderate obstruction, and 73.8% for severe obstruction (Figure 4; Table 2). SABA was prescribed for 94.6% of patients with mild airway obstruction, 82% with moderate obstruction, and 61.9% with severe obstruction (data not shown). LAMA was prescribed for 10.9% of patients with moderate airway obstruction and for 52.4% of patients with severe obstruction. LABA/ICS was prescribed for 50% of patients with severe airway obstruction (Figure 4).

### Bivariate and multivariate determinants of COPD-related healthcare use

Bivariate and multivariate determinants influencing COPD-related healthcare use are presented in Table 3. Independent variables included in the multivariate logistic analyses were age, sex, smoking status, household income, residential area, severity of airway obstruction, presence of comorbidities, and self-reported physician diagnosis of COPD. The determinants of COPD-associated healthcare use in participants with obstructive lung disease were advanced age, severe airflow limitation, presence of comorbidities, and self-reported physician diagnosis of COPD.

**Table 3:** Bivariate and multivariate determinants of COPD-related healthcare use

**Variables**	**COPD-related healthcare utilization**				
	**User**	**Non-user**			
	**No. (%)**	**No. (%)**			
	**244 (27.2)**	**653 (72.8)**	**OR**	**95% CI**	***P *** **value**
Age (yr)					
40 ~ 59	50 (19.2)	211 (80.8)	1.00		
≥60	194 (30.5)	442 (69.5)	1.56	1.03 - 2.36	0.04
Sex					
Female	74 (27.5)	195 (72.5)	1.00		
Male	170 (27.1)	458 (72.9)	1.13	0.71 - 1.77	0.61
Smoking status					
Never	75 (25.7)	217 (74.3)	1.00		
Former	82 (27.4)	217 (72.6)	1.04	0.63 - 1.70	0.89
Current §	87 (28.9)	214 (71.1)	1.32	0.82 - 2.13	0.26
Residential area					
Urban	138 (25.9)	394 (74.1)	1.00		
Rural	106 (29.0)	259 (71.0)	1.07	0.78 - 1.48	0.68
Household income					
≥Lower quartile	129 (23.5)	421 (76.6)	1.00		
<Lower quartile	115 (33.1)	232 (66.9)	1.32	0.94 - 1.85	0.10
Severity of airway obstruction					
Mild	74 (20.3)	291 (79.7)	1.00		
Moderate	128 (27.2)	342 (72.8)	1.54	1.10 - 2.15	0.01
Severe¶	42 (67.7)	20 (32.3)	6.68	3.60 - 12.38	<0.01
No. of co-morbidities					
0	107 (22.4)	370 (77.6)	1.00		
≥1	137 (32.6)	283 (67.4)	1.44	1.03 - 2.01	0.03
Self-reported physician diagnosis of COPD					
No	224 (25.8)	646 (74.3)	1.00		
Yes	20 (74.1)	7 (25.9)	5.30	2.08 - 13.54	<0.01

OR = odds ratio; CI = confidence interval. § Comparison with never smoker; ¶ Comparison with mild airway obstruction.

## Discussion

In this study, patterns of healthcare use in subjects with COPD were assessed based on representative, nationwide epidemiological cohort data combined with Korean NHI claims data. The NHI system provides an exact and reliable computerized database for all healthcare service use for the entire population of South Korea. A few epidemiological reports on COPD-related healthcare use exist in the literature [10,13-16]; however, reports on the association between healthcare use patterns and COPD severity are scarce. Our study also identified a total 27.2% (N = 244) COPD-related healthcare use rate. We observed an increase in use according to disease severity, which was a strong predictor of future healthcare use. However, PFTs were performed in only 27.5% of patients with COPD. Medications prescribed for COPD in Korea were mainly oral theophylline (44.7%) and oral corticosteroids (21.7%), regardless of the disease severity. The major determinants for COPD-related healthcare use were advanced age, disease severity, presence of comorbidities, and physician diagnosis of COPD. These factors should be considered in health plans and in disease management programs for COPD.

The present study showed that total prevalence of COPD based on PFTs was 13.4%. The prevalence of COPD in other countries varies from 7.8% to 19.7% [17-19]. In our study, the percentage of self-reported physician-diagnosed COPD was 3% (N = 27) and the percentage of PFT-based undiagnosed airway obstruction was 13.1% (N = 870). These findings are similar to the U.S. NHANES III study published by Coultas et al. [20], in which the percentage of doctor-diagnosed COPD was 3.1% (N = 179) and of undiagnosed airflow obstruction was 12.0% (N = 688). Other national data reveal that <6% of the adult population has been told by a healthcare provider that they have COPD [4,6,7,9]. The reasons for underdiagnosis of COPD include low usage of PFTs [21,22], lack of awareness of COPD, poor physician adherence to guidelines [23], and lack of educational programs on COPD. Asymptomatic COPD patients do not tend to use healthcare services. Most primary care physicians diagnosed COPD based on patients’ symptoms and smoking history [21]. Objective diagnosis using PFTs tends to produce higher prevalence estimates than patient-reported diagnosis. The underdiagnosis of COPD is likely to result in substantial morbidity and mortality.

The Global Initiative for Chronic Obstructive Lung Disease (GOLD) recommends that PFTs be used for the clinical diagnosis of COPD to avoid underdiagnosis, and to ensure proper evaluation of the severity of the airflow obstruction [4]. Other studies also reported that PFTs were underused in detecting patients with COPD and confirming the diagnosis of COPD [24,25]. Dyspnea, coughing, wheezing, and smoking history are not specific to COPD [24]. PFTs can detect asymptomatic COPD early, which allows for risk factors to be modified (i.e., smoking cessation) [25]. Early detection of COPD is important in Korean men because of that country’s high male smoking rate relative to the female smoking rate (60.6% vs. 5.2%, respectively) [26]. Han et al. revealed that only 32% of COPD patients in the United States had PFTs performed within 2 years before, or 6 months after, diagnosis [27]. Our study suggested that the low rate of PFTs performed (27.5%) might be associated with the underdiagnosis of COPD in Korea.

Interestingly, the percentages of prescribed oral theophylline (44.6%) and oral corticosteroids (21.7%) were high regardless of the severity of airflow obstruction in our study (data not shown). The high prescribing rate of oral theophylline may be associated with patients’ preference for oral medications in Korea. The low prescribing rate of LAMA and LABA/ICS may be related to underdiagnosis of COPD. The GOLD guidelines clearly outline which classes of COPD medications should be prescribed according to disease severity. A few reports demonstrate that COPD treatment guidelines reduce healthcare use and mortality [28]. Nevertheless, physician adherence to prescribing guidelines and patient adherence to drug therapy remain poor [10,29]. The COPD Resource Network Needs Assessment Survey reported that 54% of primary physicians and 94% of pulmonologists under-prescribed recommended pharmacological treatments despite acknowledging awareness of the current COPD treatment guidelines [30]. Park et al. showed that 56.7% of primary care physicians in Korea knew about the GOLD guidelines and 61.8% had spirometry equipment. However, PFTs were often underused because of problems with spirometry equipment, refusal by patients, and unawareness of the importance of screening patients for COPD. Common respiratory medications prescribed for stable COPD included oral theophylline (24.9%), inhaled anticholinergics (22.1%), oral β2-agonists (17.1%), and inhaled β2 agonists (15.7%), which was consistent with our results [31].

Our study revealed that the severity of the airflow obstruction was one of the major determinants of COPD-related healthcare use. Some authors have suggested that comorbidities or symptoms were more relevant determinants than the severity of the airway obstruction in determining healthcare use [16,32-34]. Other authors showed that the severity of COPD was strongly correlated with healthcare use [29,35]. In our study, subjects with moderate and severe airway obstruction used healthcare services more frequently than patients with mild obstruction. Additionally, in our study, age and comorbidities were also identified as determinants of COPD-related healthcare use [32]. Unlike other studies, household income [15] and smoking status [16] were not related to healthcare use. In this study, self-reported physician-diagnosed COPD was also a major determinant of healthcare use. Lack of knowledge about COPD may affect the use of healthcare, but further study is needed. However, we did not include acute exacerbation events with the classical criteria of acute exacerbation. In those criteria, acute exacerbation is defined by an acute event characterized by a worsening of the patient’s respiratory symptoms that is beyond normal day-to-day variations, and leads to a change in medication. In most studies concerned with acute exacerbation of COPD, moderate to severe exacerbation of COPD is defined by an emergency room visit or admission due to COPD. We analyzed data regarding COPD-related admissions to hospital and visits to emergency departments, which can be considered as involving moderate to severe exacerbation of COPD.

There are several limitations to our study. First, the follow-up period was only 1–3 years. Second, results from this study cannot be generalized to the entire Korean population over 40 years of age. Participants in this study might have been relatively healthy, while sedentary patients with severe airway obstruction might not have been included in our study. Thus, we cannot entirely rule out selection bias. In addition, healthcare users comprised a small sample size. Further studies need a larger, nationwide sample with a much longer follow-up period. Third, eligible participants were selected using a fixed FEV_1_/FVC ratio, and airflow obstruction was determined without consideration of the decrease in pulmonary function that occurs with age. COPD among the elderly in our study seemed over-represented [36]. Furthermore, lung function data from this study were based on pre-bronchodilator PFTs, and the inadvertent inclusion of asthma patients might have skewed the results. In addition, in the survey questionnaire, we had questions regarding self-reported physician diagnosis of asthma. In doing so, we were trying to exclude asthma patients, but it is not possible to do this using self-reported physician diagnosis criteria. It is a limitation of nationwide surveys, and it is not possible to differentiate COPD patients from asthma patients in the KNHANES study. We could not exclude from this study population asthma patients older than 40 years of age who had airflow limitation. As a result, we analyzed patterns and determinants of healthcare use in patients with airway obstruction, which might have included cases with COPD or bronchial asthma. We tried to clarify data from COPD-related ICD-10 codes in NHI claims during the 3-year follow-up period. Therefore, the number of patients who did not use healthcare resources might have included cases with COPD or asthma. Fourth, identification of patients based on insurance claims data also relies on the correct use and input of codes into the computerized insurance database. It would have been useful to have data regarding non-pharmacological interventions such as smoking cessation, physical activity, rehabilitation, and vaccination. Unfortunately, we could not extract information about non-pharmacological interventions using COPD-related claims codes from the NHI claims data, because non-pharmacological interventions are not covered and reimbursed by the NHI system in Korea. This is also a limitation. Last, we could not analyze data about respiratory symptoms because major questions about such symptoms were not included in the survey questionnaire. We accept that the absence of analysis regarding respiratory symptoms is a flaw. These factors could influence major determinants of healthcare use [16]. There is still debate about the use of spirometry vs. symptom questionnaires [37] in the screening and diagnosis of COPD. Future studies should analyze the severity of airflow obstruction with well-established lung function questionnaires.

## Conclusions

Among patients with COPD, 27.2% used COPD-related healthcare services. Only 27.5% of the 244 participants with airway obstruction who used COPD-related healthcare underwent PFTs. The percentage of prescribed medications associated with COPD increased in accordance with the severity of the COPD. However, oral theophylline and oral corticosteroids were mainly prescribed. The determinants of COPD-associated healthcare use in subjects with obstructive lung disease were advanced age, severe airflow limitation, presence of comorbidities, and self-reported physician diagnosis of COPD.

## Abbreviations

KNHANES: Korean National Health and Nutritional Examination Survey; NHI: National health insurance; COPD: Chronic obstructive pulmonary disease; PFT: Pulmonary function test; SABA: Inhaled short-acting β-agonist; LAMA: Inhaled long-acting anticholinergic; LABA/ICS: Inhaled long-acting β-agonist combined with corticosteroid; ICS: Inhaled corticosteroid.

## Competing interests

The authors declare that they have no competing interests.

## Authors’ contributions

KC, JK, and YK contributed to the study design, data analysis and interpretation, and writing of this manuscript. KK, JJ, KO, YO, and SK contributed to the study design, data analysis and interpretation, and review of this manuscript. JK and YK contributed equally to this article. All authors read and approved the final manuscript.

## Pre-publication history

The pre-publication history for this paper can be accessed here:

http://www.biomedcentral.com/1471-2466/14/27/prepub
